# Surgical management of delayed air leakage following radiofrequency ablation for non-small cell lung cancer: a case report

**DOI:** 10.3389/fradi.2026.1815270

**Published:** 2026-09-23

**Authors:** Luca Frasca, Ilaria Suriano, Antonio Sarubbi, Filippo Longo, Vincenzo Canalella, Eliodoro Faiella, Rosario Francesco Grasso, Pierfilippo Crucitti

**Affiliations:** 1 Department of Thoracic Surgery, Fondazione Policlinico Universitario Campus Bio-Medico, Rome, Italy; 2 PhD Doctor in Microbiology, Immunology, Infectious Diseases, and Transplants (MIMIT), University Tor Vergata, Rome, Italy; 3 Fellowship in Department of Thoracic and Vascular Surgery, Avicenne Hospital, Paris, France; 4 Master’s Degree Program in Medicine and Surgery, Campus Bio-Medico University, Rome, Italy; 5 Department of Radiology and Interventional Radiology, Fondazione Policlinico Universitario Campus Bio-Medico, Rome, Italy

**Keywords:** biologic sealant, delayed pneumothorax, interventional radiology, non-small cell lung cancer, persistent air leak, radiofrequency ablation, UVATS

## Abstract

We report the case of a 68-year-old male with a history of urothelial carcinoma and severe emphysema who developed a delayed pneumothorax following CT-guided radiofrequency ablation (RFA) of a pulmonary squamous cell carcinoma. Conservative management with chest drainage failed to resolve the air leak. Surgical revision via uniportal video-assisted thoracic surgery (UVATS) allowed identification of a necrotic subpleural lesion with active air leakage. Hemostatic and sealing agents (Hemopatch® and Tisseel®) were applied with long-term resolution of the complication. This case underscores the importance of surgical intervention in selected cases of persistent air leak post-RFA and the potential efficacy of biologic sealants when resection is not feasible.

## 1 Introduction

Surgical procedures are often impeded by bleeding and/or leakage of body fluids. These complications cannot always be resolved by conventional surgical techniques. Biologic sealants and hemostatic agents efficiently establish hemostasis and sealing in a variety of surgical specialties, including thoracic surgery. They are largely used to reduce prolonged air leaks and shorten the use of air drainage tube after lung surgery in patients at high risk for prolonged air leaks (1). Among these, Hemopatch®, a collagen-based patch coated with N-hydroxysuccinimide functionalized polyethylene glycol (NHS-PEG) (2), and Tisseel®, a fibrin sealant mimicking the final steps of coagulation (3), offer complementary mechanisms of action. Their combined use can effectively achieve airtight closure in high-risk pulmonary settings, such as necrotic lesions or ablation-related defects, and may obviate the need for resection in selected patients. This case illustrates the successful application of both agents to manage a delayed, refractory air leak following radiofrequency ablation (RFA), underscoring their potential role in salvage strategies.

## 2 Case presentation

A 68-year-old male, former smoker with a 45 pack-year history, was undergoing follow-up for urothelial carcinoma of the bladder (pT2) with lymph node involvement and suspected pulmonary metastases. He received maintenance therapy with Avelumab. Immunohistochemical analysis showed PD-L1 expression ranging from 25%–30%.

In 2023, surveillance high-resolution chest CT (1-mm slice thickness with multiplanar reconstructions) demonstrated interval growth of a left upper lobe subpleural nodule from 11 mm to 16 mm. Pulmonary function tests revealed severe obstructive ventilatory impairment with FEV1 1.32 L (48% predicted), FEV1/FVC ratio 54%, and DLCO 42% predicted, indicating markedly reduced diffusion capacity despite relatively limited radiologic emphysema. CT-guided biopsy confirmed primary squamous cell carcinoma (p40+, TTF-1 + in rare cells, GATA3-). Multidisciplinary tumor board discussion concluded that lobectomy posed high perioperative risk due to limited pulmonary reserve prompting a recommendation for percutaneous radiofrequency ablation (RFA) as the next step in management.

Pulmonary consultation prior to the procedure identified a high risk due to underlying interstitial lung disease. Corticosteroid therapy and bronchoalveolar lavage (BAL) were recommended. BAL was positive for *Haemophilus influenzae*, which was treated appropriately.

RFA using a Cambridge 3 cm needle×17 G, for a 12 min treatment at 98 W, 71 kJ was performed without immediate complications. Immediate post-procedural CT demonstrated the expected circumferential ground-glass halo surrounding the ablation zone without evidence of pneumothorax.

Four weeks later, the patient presented to the emergency department with acute severe dyspnea. Chest CT revealed a left-sided pneumothorax with associated subcutaneous emphysema (Figure 1**)**. A chest tube was placed in the 5th intercostal space and connected to continuous suction. Despite 12 days of drainage, persistent bubbling indicated ongoing air leak with incomplete lung re-expansion.

**Figure 1:**
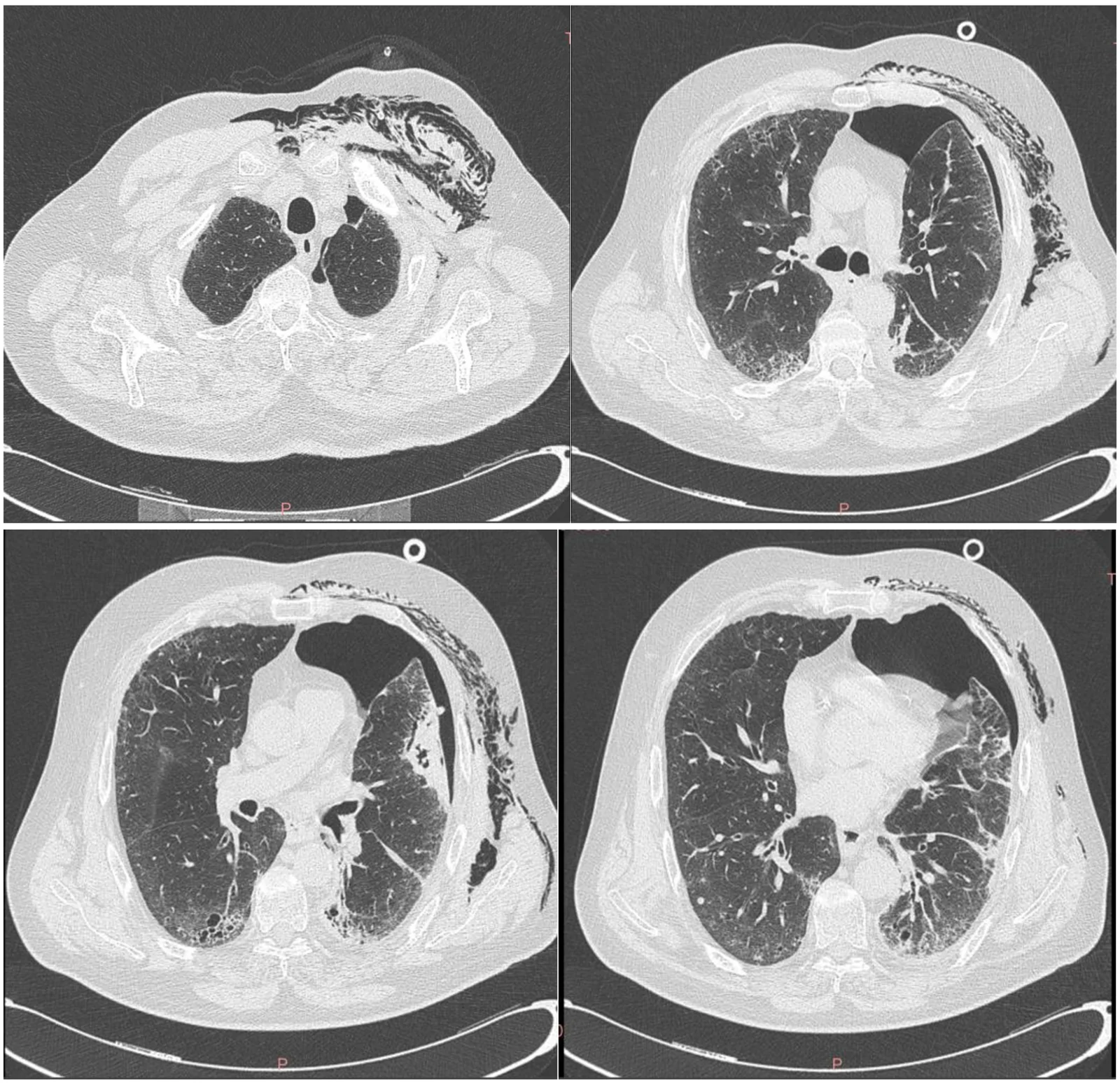
Chest CT scan performed in the emergency department demonstrating a severe left-sided pneumothorax associated with extensive subcutaneous emphysema. The axial view highlights near-complete collapse of the left lung with significant air accumulation in the pleural space. Air tracking along the chest wall soft tissues is clearly visible. These findings are consistent with acute respiratory compromise requiring urgent management.

Under general anesthesia with double-lumen intubation and lung-protective ventilation strategy (18), uniportal video-assisted thoracic surgery revealed a 1.5 cm necrotic subpleural parenchymal defect corresponding to the ablation site. Pneumatic testing confirmed active air leakage (Figure 2**)**.

**Figure 2:**
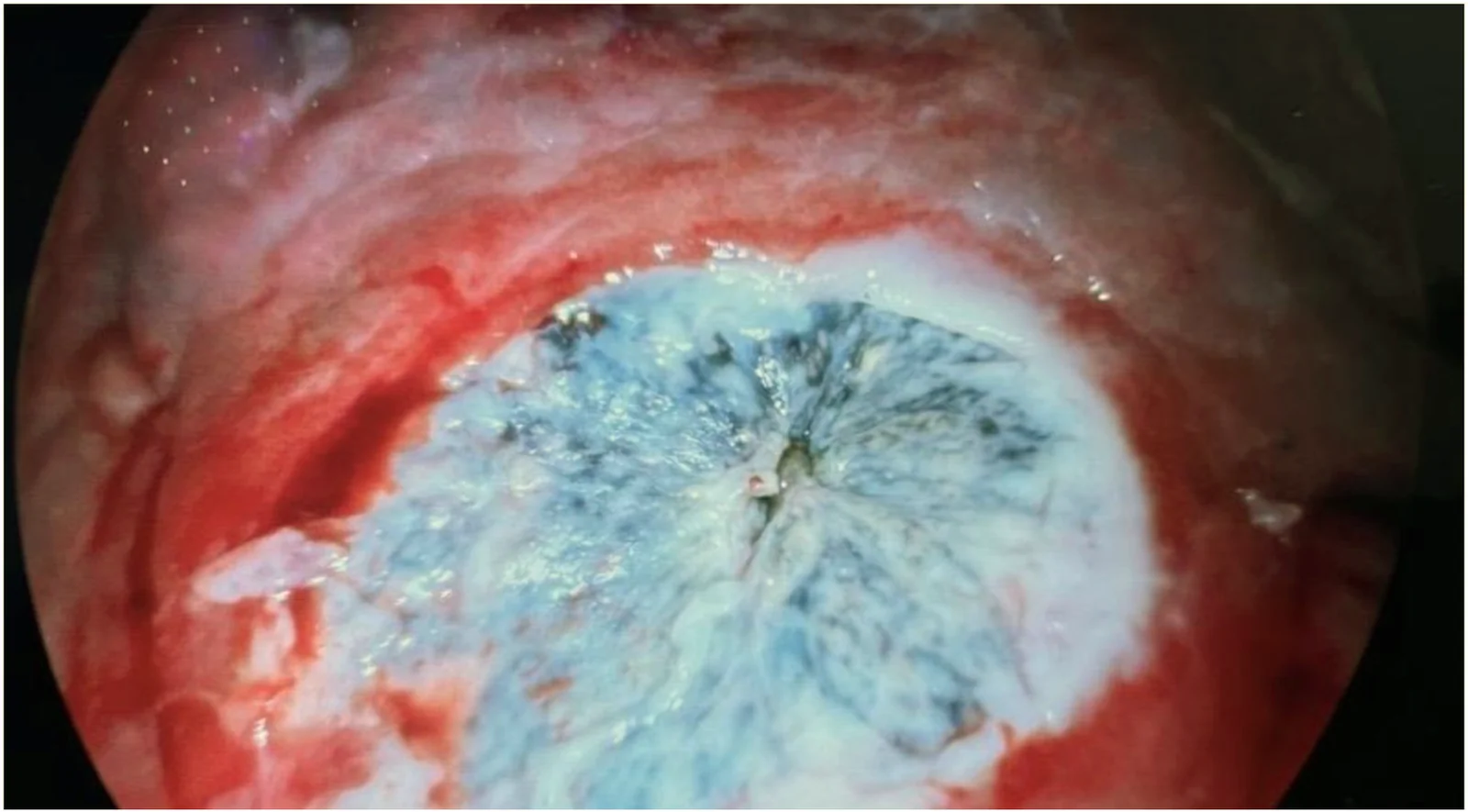
Intraoperative picture showing a 1.5 cm subpleural lesion surrounded by necrotic area in the previously ablated area of the left upper lobe. A pneumatic test was performed and confirmed the air leak at this level.

No central bronchopleural fistula involving segmental bronchi was identified.

The surrounding emphysematous parenchyma was markedly friable. Given borderline pulmonary reserve and risk of additional staple-line air leaks, wedge resection was deemed unsafe. A Hemopatch® collagen sealant was applied directly over the lesion, followed by Tisseel® both centrally and along the lesion margins to ensure sealing (Figure 3**)**. Multiple parietal pleural biopsies were taken and submitted for histologic and microbiological analysis. A 20 Fr chest tube was placed apically and connected to digital chest drainage system. Low-pressure re-expansion confirmed absence of residual air leak.

**Figure 3:**
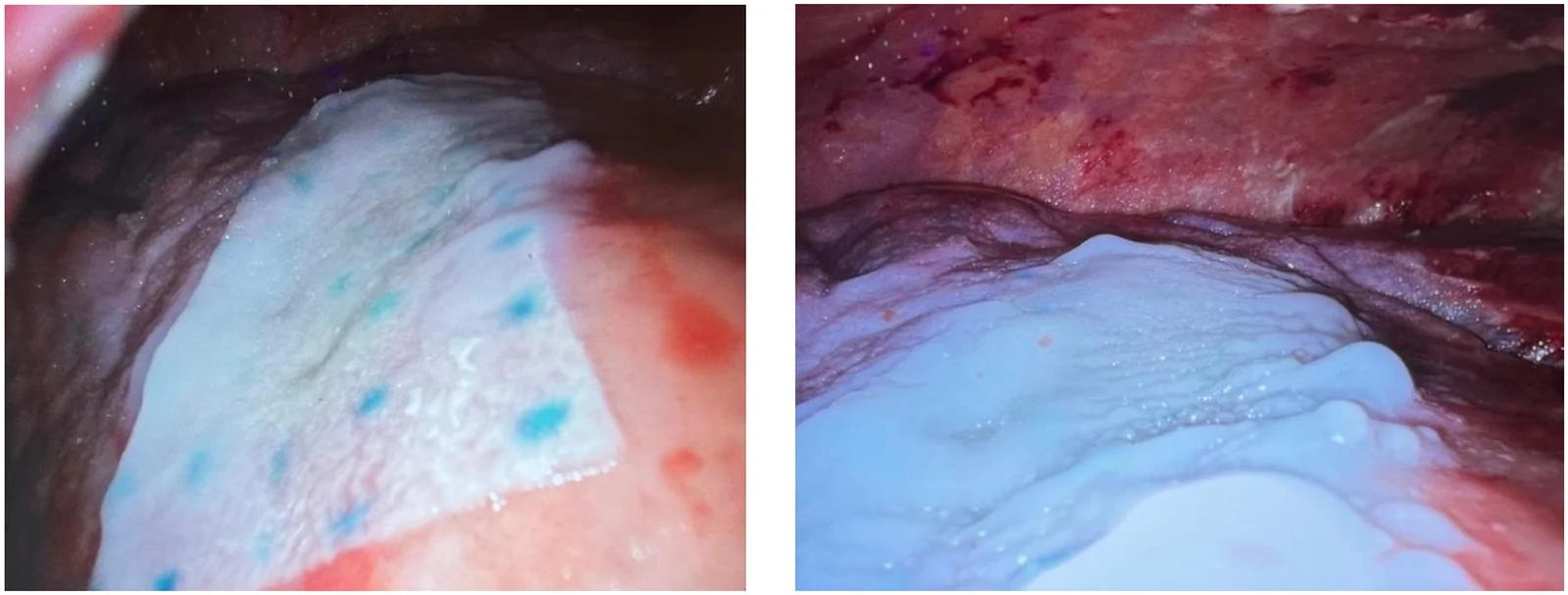
During UVATS, Hemopatch® and Tisseel® were applied to completely cover both the parenchymal lesion and its margins. Low-pressure lung re-expansion was performed, and no air leak was visible during the pneumatic test.

Postoperatively, no residual air leak was observed. A one-way valve was attached on postoperative day 5, and the patient was discharged on day 10.

Histological analysis revealed fibrotic tissue and fibrinous exudate with chronic inflammatory infiltrates. Follow-up imaging at two and four months showed no recurrence of pneumothorax, resolution of subcutaneous emphysema, and signs of localized post-ablation consolidation with a small air bubble but no active air leak (Figure 4).

**Figure 4:**
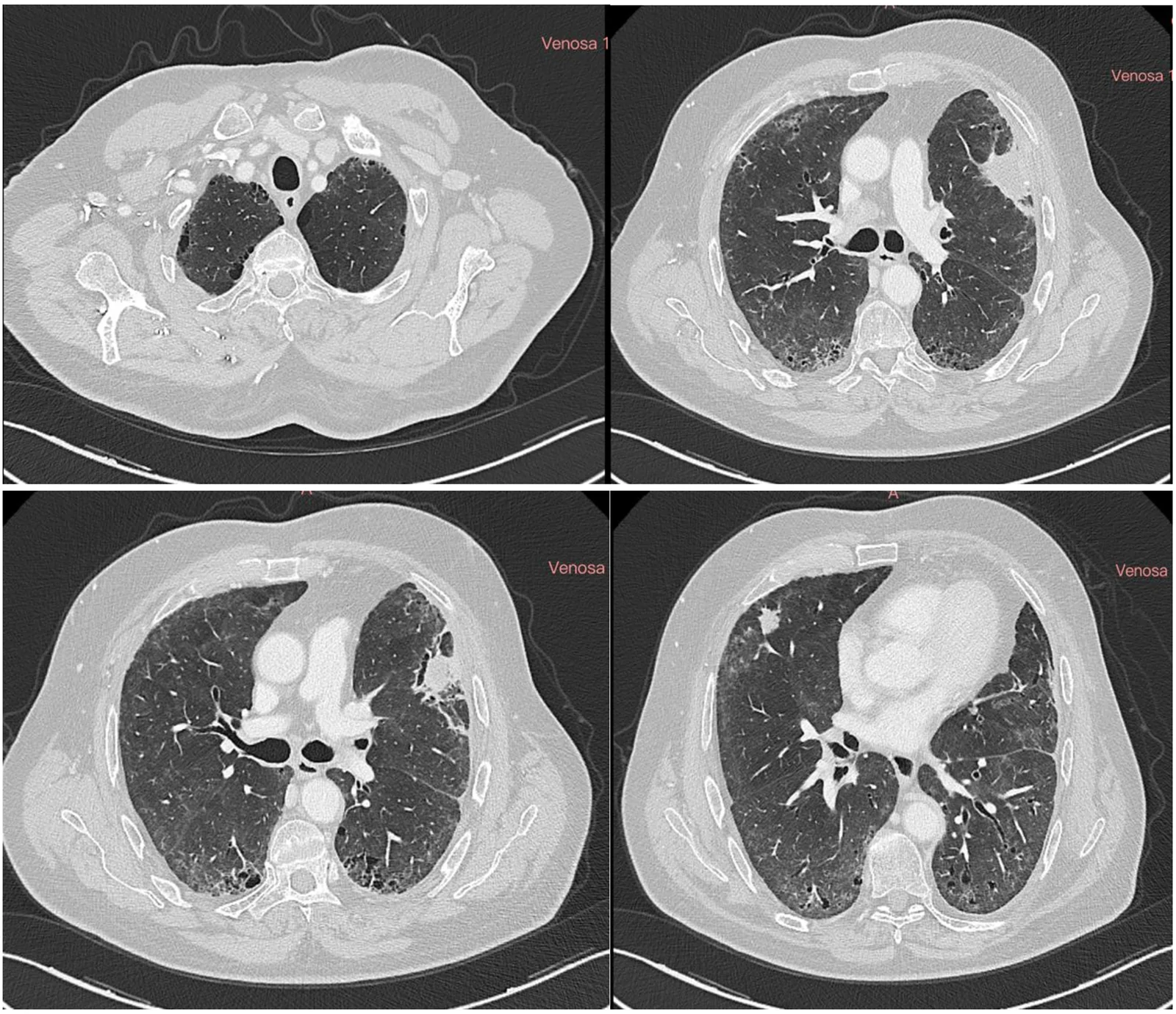
Follow-up chest CT scan demonstrating complete resolution of the previously observed pneumothorax and subcutaneous emphysema. No residual pleural air collections are visible, and normal lung re-expansion is evident. The chest wall soft tissues show full reabsorption of prior emphysematous changes. A focal area of parenchymal consolidation persists at the site of the prior ablation. This finding is consistent with post-procedural changes and reflects the expected radiological sequelae of thermal ablation.

All procedures performed in this study were in accordance with the ethical standards of the institutional and/or national research committee(s) and with the Helsinki Declaration. Written informed consent was obtained from the patient for publication of this case report. This case report was prepared in accordance with the CARE (CAse REport) guidelines.

## 3 Discussion

The detection of early-stage lung cancer has increased, also supported by lung cancer screening programs, particularly non-small cell lung cancer (NSCLC), with squamous cell carcinoma being a significant subtype (4, 5). While surgical resection remains the gold standard for operable NSCLC, comorbidities such as chronic obstructive pulmonary disease (COPD), interstitial lung disease (ILD), or poor general condition may contraindicate surgery (6). In these high-risk patients, percutaneous RFA has emerged as a valid option for localized lung disease in the absence of nodal involvement (7–9). The National Comprehensive Cancer Network (NCCN) guidelines recognize RFA as an acceptable treatment option in such cases (10).

RFA relies on thermal coagulation necrosis, exploiting the low impedance of air-filled lung tissue to localize damage within the tumor, effectively giving the outcomes of R0 resection in suitable candidates (11, 12).

The most frequent complication of pulmonary RFA is pneumothorax, with incidence rates ranging between 10%–60% (13). Other short-term complications include pleural effusion and pulmonary hemorrhage. Rare but reported adverse events encompass pneumonia, bronchopleural fistula (BPF), pleuritis, lung abscess, gas embolism, rib or vertebral injury, pericardial effusion, cardiac arrhythmias and myocardial infarction (14). Delayed complications, such as prolonged air leaks or subacute necrosis-related pneumothorax, are sparsely documented. While BPF remains the only delayed complication consistently reported in the literature (15), no cases describing delayed air leak due to progressive post-ablation necrosis in the absence of fistulization have been documented.

Persistent air leaks typically respond to conservative measures, including chest drainage and pleurodesis (16). However, the effectiveness of chemical pleurodesis may be limited when adequate pleural apposition cannot be achieved, particularly in the presence of incomplete lung re-expansion and a substantial ongoing air leak. In the present case, the persistence of the air leak despite prolonged chest drainage and the presence of a focal post-ablation parenchymal defect supported surgical exploration, allowing direct identification and control of the source of the leak while avoiding formal parenchymal resection. In our case, tissue necrosis evolved several weeks post-RFA and resulted in a delayed-onset air leak refractory to conservative therapy. Surgical intervention, including parenchymal resection, is reserved for cases with a prolonged air leak, underlying infection, or failure of conservative strategies (17). Minimally invasive approaches could allow targeted treatment with reduced postoperative morbidity and mortality.

Hemopatch® is a flexible, resorbable collagen pad derived from bovine dermis and coated with a reactive NHS-PEG (N-hydroxysuccinimide functionalized polyethylene glycol) layer (2). Upon contact with moist tissue, the NHS-PEG component forms covalent bonds with proteins, creating a rapid and strong mechanical seal, while the collagen matrix promotes platelet adhesion and supports hemostasis. Tisseel®, on the other hand, is a two-component fibrin sealant composed of human-derived fibrinogen and thrombin (3). Once mixed and applied to tissue, it replicates the final steps of coagulation, forming a stable fibrin clot that adheres to the surface and supports sealing of air or fluid leaks. When used together, Hemopatch® provides structural stability and surface adherence, while Tisseel® reinforces the seal and penetrates tissue irregularities. In our case, this synergistic application allowed effective closure of a necrotic subpleural laceration, avoiding high-risk resection and achieving durable resolution of the air leak. These properties support their use not only as adjuncts but as primary tools in the management of complex pulmonary air leaks, particularly in high-risk patients with impaired healing potential.

In cases where parenchymal resection is unfeasible, due to lesion proximity to the hilum, the presence of necrotic tissue or the risk of functional loss, biological sealants could offer an effective alternative. Their application achieved immediate cessation of air leak and maintained parenchymal integrity without requiring resection. This combination, although well documented in thoracic surgery, is not described in the context of RFA-related complications.

## 4 Conclusions

This case highlights a previously unreported delayed complication of RFA in the form of a persistent subpleural air leak, underscoring the need for long-term vigilance post-procedure. It demonstrates the value of a multidisciplinary approach and the utility of UVATS in managing non-resolving pneumothorax. Importantly, it emphasizes the effectiveness of biologic sealants when anatomical resection is not feasible and the technical relevance of low-pressure lung re-expansion. In appropriately selected patients, this conservative-surgical hybrid approach may provide definitive treatment while preserving lung function.

## Data Availability

The raw data supporting the conclusions of this article will be made available by the authors, without undue reservation.

## References

[1] Malapert G Hanna HA Pages PB Bernard A. Surgical sealant for the prevention of prolonged air leak after lung resection: meta-analysis. Ann Thorac Surg. (2010) 90:1779–85. 10.1016/j.athoracsur.2010.07.033 21095309

[2] Olthof PB Rassam F Van Gulik TM. The use of a NHS-PEG coated, collagen-based sealant in a patient undergoing associating liver partition and portal vein ligation for staged hepatectomy (ALPPS). Int J Surg Case Rep. (2018) 47:7–10. 10.1016/j.ijscr.2018.03.038 29698885 PMC5994687

[3] Grunenwald D Vouhé PR Neveux JY The use of fibrin sealant (tissucol/tisseel) in thoracic surgery. In: Fibrin Sealant in Operative Medicine; Schlag G Redl H, Edn.; Springer: Berlin/Heidelberg, Germany, (1986): p. 63–74. 10.1007/978-3-642-71633-1_6

[4] Chen CL Hsu JS Shen YW Hsu CH Kao SY Lai WA. Changes in staging and management of non-small cell lung cancer patients following the implementation of low-dose chest computed tomography screening at Kaohsiung medical university hospital. Cancers (Basel). (2024) 16:3727. 10.3390/cancers16223727 39594683 PMC11592150

[5] Sabbula BR Gasalberti DP Mukkamalla SKR Anjum F. Squamous cell lung cancer. In: StatPearls [Internet]. Treasure Island (FL): StatPearls Publishing (2026). Available online at: https://www.ncbi.nlm.nih.gov/books/NBK564510/ (Accessed February 14, 2024). 33232091

[6] Kim H Yoo H Pyo H Ahn YC Noh JM Ju SG. Impact of underlying pulmonary diseases on treatment outcomes in early-stage non-small cell lung cancer treated with definitive radiotherapy. Int J Chron Obstruct Pulmon Dis. (2019) 14:2273–81. 10.2147/COPD.S210759 31631997 PMC6782029

[7] Zhao Q Wang J Fu YL Hu B. Radiofrequency ablation for stage < IIB non-small cell lung cancer: opportunities, challenges, and the road ahead. Thorac Cancer. (2023) 14:3181–90. 10.1111/1759-7714.15114 37740563 PMC10643797

[8] Suriano I Frasca L Longo F Sarubbi A Tacchi G Crucitti P. Diagnostic yield of CE-EBUS in mediastinal and hilar lymphadenopathy: a preliminary study. J Clin Med. (2025) 14:2800. 10.3390/jcm14082800 40283633 PMC12027627

[9] Schrevens L Lorent N Dooms C Vansteenkiste J. The role of PET scan in diagnosis, staging, and management of non-small cell lung cancer. Oncologist. (2004) 9:633–43. 10.1634/theoncologist.9-6-633 15561807

[10] Ambrogi MC Fanucchi O Cioni R Dini P De Liperi A Cappelli C. Long-term results of radiofrequency ablation treatment of stage I non-small cell lung cancer: a prospective intention-to-treat study. J Thorac Oncol. (2011) 6:2044–51. 10.1097/JTO.0b013e31822d538d 22052222

[11] Goldberg SN Gazelle GS Mueller PR. Thermal ablation therapy for focal malignancy: a unified approach to underlying principles, techniques, and diagnostic imaging guidance. AJR Am J Roentgenol. (2000) 174:323–31. 10.2214/ajr.174.2.1740323 10658699

[12] Yuan Z Wang Y Zhang J Zheng J Li W. A meta-analysis of clinical outcomes after radiofrequency ablation and microwave ablation for lung cancer and pulmonary metastases. J Am Coll Radiol. (2019) 16:302–14. 10.1016/j.jacr.2018.10.012 30642784

[13] Li G Xue M Chen W Yi S. Efficacy and safety of radiofrequency ablation for lung cancers: a systematic review and meta-analysis. Eur J Radiol. (2018) 100:92–8. 10.1016/j.ejrad.2018.01.009 29496085

[14] Hiraki T Gobara H Fujiwara H Ishii H Tomita K Uka M. Lung cancer ablation: complications. Semin Intervent Radiol. (2013) 30:169–75. 10.1055/s-0033-1342958 24436533 PMC3709965

[15] Sakurai J Hiraki T Mukai T Mimura H Yasui K Gobara H. Intractable pneumothorax due to bronchopleural fistula after radiofrequency ablation of lung tumors. J Vasc Interv Radiol. (2007) 18:141–5. 10.1016/j.jvir.2006.10.011 17296716

[16] Dugan KC Laxmanan B Murgu S Hogarth DK. Management of persistent air leaks. Chest. (2017) 152:417–23. 10.1016/j.chest.2017.02.020 28267436 PMC6026238

[17] Mundanchira G Frye L Reisenauer J Agrawal A. The persistent problem of persistent air-leaks: approach to management. Curr Opin Pulm Med. (2025) 31:28–34. 10.1097/MCP.0000000000001134 39635887

[18] Guay J Ochroch EA. Intraoperative use of low volume ventilation to decrease postoperative mortality, mechanical ventilation, lengths of stay and lung injury in patients without acute lung injury. Cochrane Database Syst Rev. (2015) 12: CD011151. 10.1002/14651858.CD011151.pub2 26641378

